# Functional MRI-based study of emotional experience in patients with psychogenic non-epileptic seizures: Protocol for an observational case-control study–EMOCRISES study

**DOI:** 10.1371/journal.pone.0262216

**Published:** 2022-01-07

**Authors:** Pierre Fauvé, Louise Tyvaert, Cyril Husson, Emmanuelle Hologne, Xiaoqing Gao, Louis Maillard, Raymund Schwan, Claire Banasiak, Wissam El–Hage, Gabriela Hossu, Coraline Hingray

**Affiliations:** 1 Pôle Hospitalo-Universitaire de Psychiatrie d’Adultes et d’Addictologie du Grand Nancy, Centre Psychothérapique de Nancy, Laxou, France; 2 Faculté de Médecine de Nancy, Université de Lorraine, Vandœuvre-lès-Nancy, France; 3 Département de Neurologie, Centre Hospitalier Régional Universitaire de Nancy, Nancy, France; 4 Centre de Recherche en Automatique de Nancy, U7039, Centre National de la Recherche Scientifique et Université de Lorraine, Nancy, France; 5 Center for Psychological Sciences, Zhejiang University, Hangzhou, China; 6 Centre d’Investigation Clinique–Innovation Technologique, Centre Hospitalier Régional Universitaire de Nancy, Nancy, France; 7 iBrain, U1253, Institut National de la Santé et de la Recherche Médicale et Université de Tours, Tours, France; 8 Clinique Psychiatrique Universitaire, Centre Hospitalier Régional Universitaire de Tours, Tours, France; 9 IADI, U1254, Institut National de la Santé et de la Recherche Médicale et Université de Lorraine, Nancy, France; Public Library of Science, UNITED KINGDOM

## Abstract

**Background:**

Psychogenic non epileptic seizures (PNES) are a frequent, disabling and costly disorder for which there is no consensual caring. They are considered as a dissociative disorder and they share many common characteristics with post-traumatic stress disorder (PTSD). Nevertheless, their pathophysiology is still unclear. In this study, we plan to obtain new data comparing functional brain activity of participants suffering from PNES, from PTSD and healthy controls via functional brain MRI during resting state and under emotional visual stimulation. The protocol presented hereunder describes an observational study with no direct treatment implication. Nevertheless, it could lead to a better understanding of PNES and to identifying targets for specialised cares of post-traumatic or dissociative disorders, like repetitive transcranial magnetic stimulation.

**Methods & analysis:**

This is a prospective, single-centre, interventional, non-randomized, open, controlled and exploratory clinical study. It will involve 75 adult French, right-handed women in 3 groups, either suffering from PNES or PTSD, or healthy controls. An informed consent will be signed by each participant. All of them will be given psychiatric tests to assess dissociation and alexithymia, psychopathological profile and history, and emotional recognition. Each participant will undergo a functional brain MRI. We will record anatomical images and five functional imaging sequences including emotional periodic oscillatory stimulation, standard emotional stimulation, Go / No Go task under emotional stimulation, and resting state. Analysis will include a descriptive analysis of all participants and the treatment for functional magnetic resonance imaging images of each sequence.

**Registration, ethics & dissemination:**

This study was approved the regional Protection of Persons Committee under the reference 16.10.01 and by the French National Medical Security Agency under the reference 2016-A01295-46. The protocol and results will be published in peer-reviewed academic medical journals and disseminated to research teams, databases, specialised media and concerned patients’ organisations.

## Introduction

Psychogenic non epileptic seizures are an important health problem around the world [1]. They are periods of abnormal behaviour and experience typically involving impairment of consciousness, flaccid or rigid collapse and/or tremulous limb movements [2]. PNES are episodic functional neurological symptoms which superficially resemble epileptic seizures, but which are not caused by epileptic discharges in the brain [3]. It is estimated that 20–30% of patients referred to epilepsy centres with intractable seizures have been misdiagnosed and have in fact PNES [4]. PNES are one of the three commonest diagnoses among patients presenting transient loss of consciousness [5]. PNES are also the most frequent functional symptom presenting to neurologists [6]. The incidence of PNES has been evaluated to be 4.09/100,000 per year [7], which is around the same level as multiple sclerosis [8]. PNES most commonly manifest from ages 15–30 and three quarters of the patients in most series are female [9]. PNES are still poorly known to health professionals [10]. The right diagnosis is usually late compared to first symptoms (diagnosis mean delay of 7 years). It results in delay of appropriate psychological treatment [11].

PNES are associated with high medical utilization rates and high personal and societal costs [4, 12]. PNES deeply affect the patient’s quality of life owing to the effects of unnecessary anticonvulsant medication, prohibited driving, sick leave and psychiatric comorbidity [4]. The challenge of better understanding, diagnosing and treating PNES is therefore essential for health professionals, patients and society.

Despite a growing literature on PNES, their understanding is still limited with a lot of grey areas to explore. The traditional dualistic approach to the understanding of PNES has provided psychoanalytic/psychodynamic perspectives, characterizing these disorders as “medically unexplained”. Fortunately, psychosocial model of PNES with predisposing, precipitating and perpetuating factors were described [5, 13]. Experts recognize the importance of emotional dysregulation and impact of traumatic events in the etiopathogenesis of PNES [5, 14].

PNES could be considered as post-traumatic disease, like post-traumatic stress disorder (PTSD). These two mental disorders are similar on several angles. The prevalence of psychological trauma is at least 75% in patients suffering from PNES [5, 15]. They also share the same psychiatric comorbidities: anxiety disorders, major depressive disorder, somatic symptom disorders. A possible link between PTSD and PNES is alleged in the literature [1, 16]. On one hand, flashbacks of the trauma and hypervigilance would be the main symptoms (in PTSD); on the other hand, it would be somatic (motor) dissociation phenomena (in PNES). The comparison between these two clinical populations (PNES patients and PTSD patients) is interesting and could be very enlightening and informative.

PNES could be also considered as an emotional disorder. Most of patients suffering from PNES present moderate to severe alexithymia, i.e. trouble in identifying or recognising their emotions [17]. In addition, the precipitating factors of PNES occurring shortly before seizures are mostly emotional: fears, feeling of helplessness, frustration, anger or even intense joy, and studies have found profiles of emotional deregulation [18]. Patients with PNES present altered behavioural and neurovegetative responses to emotional pictures, compared to controls and patients suffering from PTSD [19]. A better understanding of brain disturbances during emotional tasks would really be a step forward in understanding this pathology.

The biological underpinnings of this disorder are less known even if there is more and more research on this subject. Brain imaging is a preferential way to explore neurobiological mechanism of PNES. Although the results of recent meta-analysis appear inconclusive, they nonetheless provide some evidence for an association between structural and functional brain abnormalities in patients with PNES, which may contribute to a biopsychosocial description of a condition often referred as “medically unexplained” [20].

Convergent neuroimaging findings implicate alterations in brain circuits mediating emotional expression, regulation and awareness (anterior cingulate and ventromedial prefrontal cortices, insula, amygdala, vermis), cognitive control and motor inhibition (dorsal anterior cingulate, dorsolateral prefrontal, inferior frontal cortices), self-referential processing and perceptual awareness (posterior parietal cortex, temporoparietal junction), and motor planning and coordination (supplementary motor area, cerebellum) [21, 22]. Hyper-connectivity between brain regions involved in emotion processing (insula) and motor function (precentral sulcus) goes unchecked by frontal brain regions involved in inhibitory control, could resulting in PNES [23, 24]. All these findings are important because they tentatively propose an underlying physical PNES substrate in the brain, which has significant implications for how we view PNES.

Nevertheless, most of the published studies were carried out in resting state without exposing patients to emotional or specific tasks. We do not know what the neurophysiologic mechanisms of the emotional experience in patients with PNES are. In these circumstances, this study would be the first brain functional imaging protocol among patients suffering from PNES, focusing on the effects of an emotional task. This new approach would investigate the mechanisms under emotional dysregulation, which is a main factor in the appearance and persistence of PNES. The challenge of a better understanding of the PNES is major and should allow new therapeutic leads.

PNES treatment is indeed difficult. By now, no drug treatment has proved effective on PNES symptoms. There is no total consensus on their care management and treatment [3, 25], even if cognitive behaviour therapy has demonstrated their effectiveness [8]. However, innovative treatments like rTMS or other neuromodulation techniques are promising tools for the treatment of PNES in clinical practice [26–28]. Specific good target for neuromodulation therapy in PNES are not yet identified. In this study, we intend to explore the pathophysiological emotional mechanisms of PNES to eventually suggest better brain structure target for neuromodulation.

### Objectives

#### Main goal & primary outcome

To compare brain activity during emotional tasks in fMRI (BOLD contrast sequences) between: patients suffering from PNES, patients suffering from PTSD and healthy controls, with age and education level matching.

#### Secondary goals & outcomes

1. To compare subjective experience rating across the three groups, during emotional pictures watching task.
2. To compare differences of cerebral functional connectivity (main physiological brain networks) in resting state fMRI across the three groups.
3. To study the correlation between cerebral activity and level of dissociation and alexithymia in PNES and PTSD groups.

One of our hypothesis is that in PNES patients’ brains, during emotional tasks, on one hand there is a hyper activation of the limbic system, which is a sign of pathological emotional experience, and on the other hand, there is an inhibition or hypo activation of the brain structures responsible for the motor control or consciousness, letting the PNES itself to show. We assume limbic hyper activation would be higher in PNES patients compared to PTSD patients, and higher in PTSD patients compared to healthy controls. We also suppose hypo activation of the motor control structures would be higher in PNES patients than in healthy controls and PTSD patients, and identical in both healthy controls and PTSD patients.

## Methods & analysis

This is a prospective, single-centre, interventional, non-randomized, open, controlled and exploratory clinical study.

### Participants

Seventy-five right-handed women will be recruited amongst patients of the university hospitals of Nancy and volunteers in Lorraineregion, and included in 3 different groups: 25 patients with PNES, 25 patients with PTSD, and 25 healthy controls.

#### Sample size

EMOCRISES is a pilot study. The 75-participants sample size cannot be calculated in a standard way because of the lack of previous data. Nevertheless, the number of subjects set is based on data from the literature. It is accepted that an fMRI study can be conducted in groups of 12 subjects with sufficient statistical power [29]. Furthermore, all fMRI studies conducted to date in PNES and PTSD have been conducted on groups of no more than 18 participants [23, 24], which nevertheless allows for a relevant statistical analysis.

### Ethics & authorizations

The study will be conducted according to the Declaration of Helsinki and received approval by local and national committees. Ethics approval has been obtained on October 6th, 2016 from the institutional research ethics board (Comité de Protection des Personnes–CPP EST III) under the reference 16.10.01. EMOCRISES was accepted by the French National Medical Security Agency (ANSM) on November 2, 2016 under the reference 2016-A01295-46. It was registered as “Emotional Lived in Patients Suffering From Psychogenic Nonepileptic Seizures: Study by Functional Magnetic Resonance Imaging (EMOCRISES)” study under ClinicalTrials identifier NCT02976545 (registered November 29^th^, 2016, https://clinicaltrials.gov/ct2/show/record/NCT02976545). A written informed consent will be obtained from each participant. Every participant will be informed of the tests and examinations they should undergo, and that they are free to withdraw their consent and stop their participation at any time before or during the MRI.

About legal registrations, see also **Trial Status**.

### Recruitment, schedule and study duration

**Fig 1:**
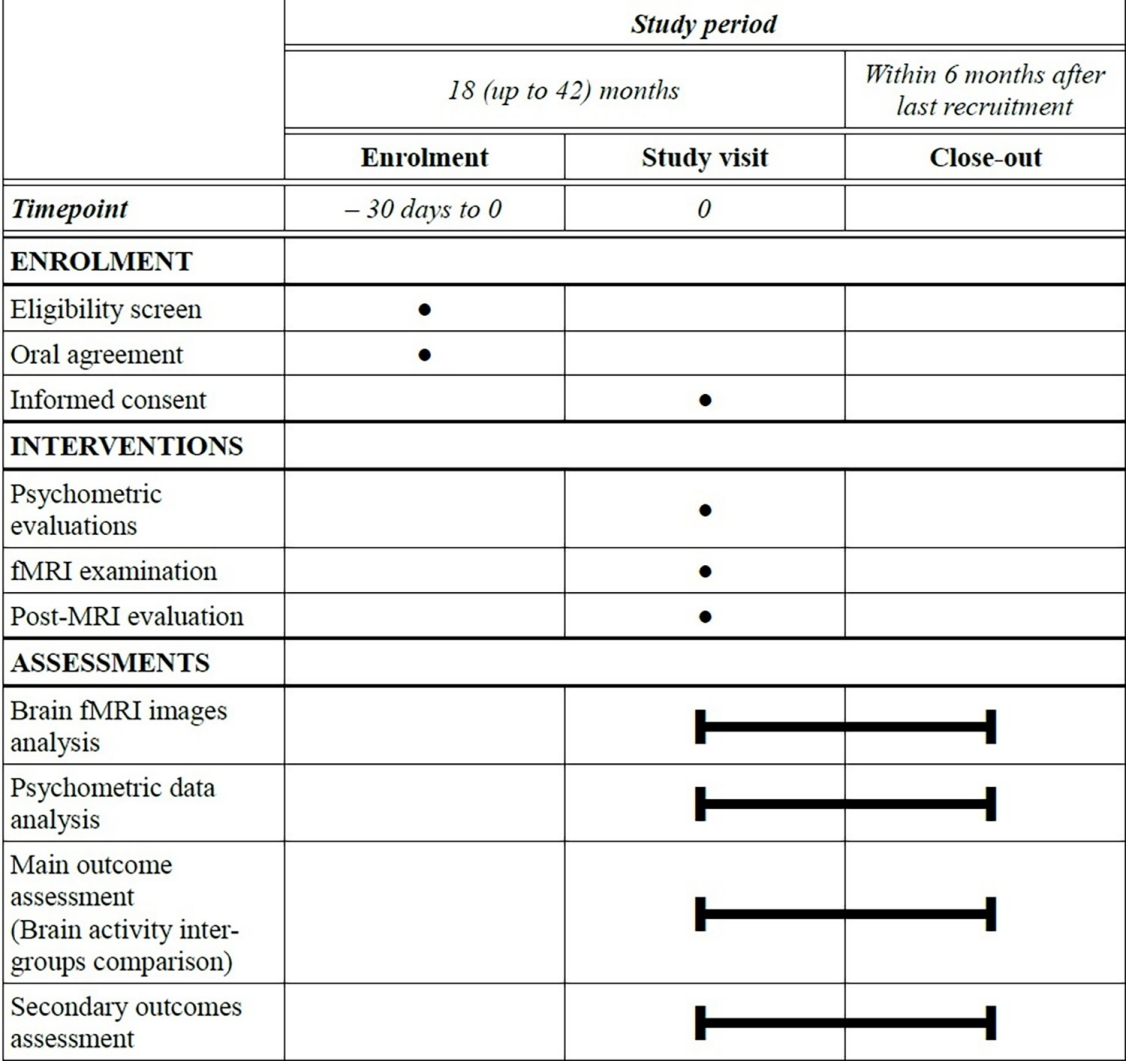
Study schedule table.

The planned total study duration is of 24 months up to 48 months depending on recruitment, including 18 to 42 months of inclusion (Fig 1). The estimated duration of participation for each participant is of 2 to 3 hours in one go, finishing after the last medical interview after the MRI examination or at the request of the participant (cf. below). No follow-up is planned in this protocol, although the participants are informed they can reach the investigators for any subsequent question regarding the study or their participation, and given the contact details to do so.

#### Inclusion criteria

- Minimal age of 18 years,
- Female,
- Right-handed,
- Affiliation to the French social security system,
- Informed signing of the study consent.

#### Inclusion criteria for PNES group

PNES co-diagnosed by a psychiatrist and a neurologist specialized in epilepsy, based on clinical symptoms analysis and EEG (electroencephalography) examination or video-EEG recording, and absence of concomitant epilepsy.

Given the difficulty to establish a positive diagnosis of PNES, alongside the exclusion criterion of epilepsy, we will apply the international recommendations for PNES diagnosis and level of certainty as synthesised by [3]. Only the two last levels of probability of PNES will conduct to potential inclusion, namely “clinically established” diagnosis and “documented” diagnosis of PNES. A clinically established diagnosis requires medical history consistent with PNES, a seizure (event with typicalsemiology) witnessed by the clinicians (on video or visually), and a seizure EEG showing no epileptiform activity. A documented diagnosis requires medical history consistent with PNES, and a video-EEG recording at the same time a seizure with typical semiology of PNES, and the corresponding EEG showing no epileptiform activity.

#### Inclusion criteria for PTSD group

PTSD diagnosed by a psychiatrist, based on clinical symptoms as recorded in DSM5.

#### Inclusion criteria for healthy controls

Healthy controls will be paired up with patients on age and level of schooling criteria.

#### Exclusion criteria

- Neurological history or comorbidity (epilepsy included),
- Current neurological treatment,
- Dosage change or introduction of psychoactive treatment dating back to less than a month,
- Psychotic comorbidity (schizophrenia),
- Addiction history apart for tobacco smoking,
- History of head trauma,
- Mental retardation,
- Being the subject of articles L.1121-5 to L.1121-8 and L1122-1-2 of the French Code of Public Health (i.e. being a not-legally-responsible adult person),
- Lack of French language proficiency or inability to understand instructions; uncorrected visual impairment; any contraindication to MRI (pacemaker, ferromagnetic foreign body, claustrophobia, etc.),
- Pregnancy.

#### Exclusion criteria for PNES group

Diagnosis of PNES challenged or denied by the patient.

#### Exclusion criteria for PTSD group

Diagnosis of PTSD challenged or denied by the patient, or current or history of functional neurological symptom disorder, PNES (conversion disorder).

#### Exclusion criteria for healthy controls

History of PTSD, PNES or other dissociative or somatic symptom disorder.

### Psychiatric evaluation

All participants will be given psychiatric tests by a psychiatrist to assess:

1. **Dissociation and alexithymia**: tests will include DES self-rated dissociation scale and TAS 20 self-rated alexithymia scale, for all subjects.
2. **Psychopathological profile**: this profile will be described by detailing ongoing and history of mental disorders detected by the MINI questionnaire (version 5.0.0 DSM-IV). Specific attention will be paid to depression, anxiety and traumatic experience. Depression will be evaluated by MADRS scale and Beck 21 self-rated evaluation. Anxiety will be assessed by HAMA clinician-rated scale and STAI self-rated questionnaire for anxiety-state. History of traumatic experiences and post-traumatic symptoms will be respectively researched via CTQ and PCL-5 self-rated questionnaires. All participants will undergo these tests.
3. **Emotional recognition**: by noting valence and intensity of emotional pictures from IAPS (International Affective Picture System) [30, 31] and NAPS (Nencki Affective Picture System) [32] databases. These emotional valence and intensity will be assessed using standard Manikin Self-Assessment scales (SAM) from 1 to 9. All participants will undergo these tests.

Despite low IQ is a known risk factor in PNES [33], we will not conduct IQ assessment on participant, in order not to complicate recruitment and protocol. This can be a limitation of the study. Concerning cognitive abilities criteria, only persons with mental retardation, illiterate or who cannot understand and follow written and oral instructions and questions of the protocol will be excluded. Besides, IQ will not be a matching factor, and the participant will be paired on age and schooling level criteria as stated above.

### MRI data acquisition

We will instruct each subject to look closely at the pictures throughout their displaying during the MRI, but we will not warn the subjects that she will have to assess emotional valence and intensity of some of the shown pictures after the examination. This, in order to avoid bias introduced by control of their emotional experience [34] or the verbal quotation [35].

We will project task stimuli onto a translucent screen by a projector placed directly outside the magnet bore. Participants will see the screen via a mirror located in the head coil. Vision correction will be performed using non-magnetic glasses, which can vary in the range of -5 to +5 with 0.5 dioptre steps. We will forward-project all stimuli onto a screen in the MRI scanner room using ePrime software (Version 3.0, Psychology Software Tools Inc., Pittsburgh, PA, US) for emotional and Go/No Go paradigm and using a homemade software for oscillatory stimulation paradigms and a calibrated projector (Panasonic PT-AE500E).

We will acquire whole brain functional MR images using a 3-Tesla magnet (Magnetom Prisma; Siemens Healthcare GmbH, Germany) with a 64-channel receive-only head coil. For all participants, high-resolution anatomical images will be acquired using a 3D Turbo Flash sequence with the following parameters: TR = 2200ms; TE = 2.93ms; TI = 900ms; flip angle 8°; in plane resolution 0.89x0.89mm2; slice thickness 1mm. We will acquire 160 volumes parallel to the anterior–posterior commissural line, covering the whole brain extending from vertex to the inferior parts of the cerebellum.

We will get BOLD gradient echo–echoplanar images (GE-EPI) during all 5 functional MRI paradigms with the following imaging parameters: TE = 30ms, 40 axial slices, slice thickness 3mm, slices order: interleaved, in plane resolution 2x2mm^2^, no interslice gap, flip angle 77°. For these functional MRI sequences, TR will be adjusted on the paradigm’s design: TR = 2500ms for emotional, Go/No Go and Resting state one; and TR = 1500ms for periodic oscillatory stimulations. Total duration of MRI acquisition will be less than one hour.

Any adverse event occurring before or during the MRI examination will be reported on a dedicated support with the date and time. If needed, examination will be terminated at any time on participant’s request or on medical decision.

Five functional imaging sequences will be conducted and are described hereinbelow:

1. Negative emotional periodic oscillatory stimulation
2. Emotional stimulation
3. Emotional Go / No Go stimulation
4. Resting state
5. Positive emotional periodic oscillatory stimulation

#### 1. Negative emotional periodic oscillatory stimulation sequence

Sequences 1 & 5 are symmetrical in structure and both are described here. They differ in content and will be processed separately during the functional MRI imaging.

We selected pictures from the IAPS, NAPS and Geneva Affective Picture Database (GAPED) [36] databases according to their emotional valence and intensity in order to make 2 sub-groups of pictures: one with positive valence and high intensity emotional pictures (respectively rated > 6 and > 5.5), and one with negative valence and high intensity emotional pictures (respectively rated < 4 and > 5.5). We excluded sex-themed pictures of negative valence. We selected as simple as possible pictures in order to let the subjects quickly identify them as positive, neutral or negative.

**Fig 2:**
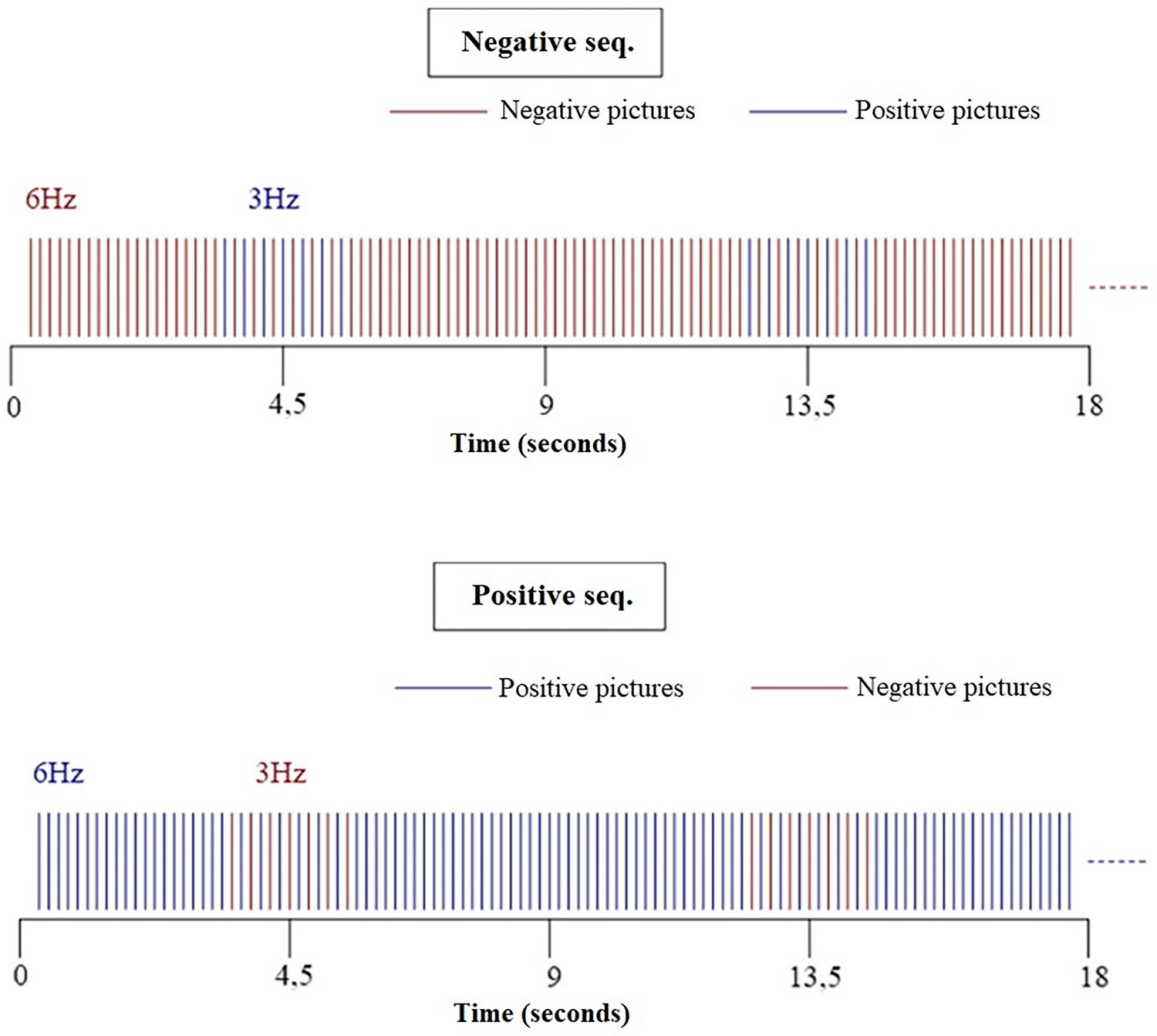
Emotional periodic oscillatory stimulation sequences schemes.

Two series of 6 minutes 33 seconds each will be shown: in the first sub-sequence (Fig 2), negative images will be displayed at a frequency of 6 Hertz (6 pictures per second). Every 9 seconds, the software will insert a series of 7 positive pictures at a frequency of 3 Hertz (3 frames per second), alternating with negative pictures. In the second sub-sequence (Fig 2), positive images will be displayed at a frequency of 6 Hertz. Every 9 seconds, a sequence of 7 images with negative valence we will inserted, at a frequency of 3 Hertz, alternating with the positive images.

Throughout this projection, a black, unmoving cross will be displayed in the centre of the screen. From time to time, this cross will turn white for a glimpse. We will ask subjects to identify each time the cross’ colour changes and indicate it with a button placed at right hand. We will use this to assess the subjects’ attention and focus on the MRI scanner and on the task.

#### 2. Emotional pictures viewing sequence

**Fig 3:**
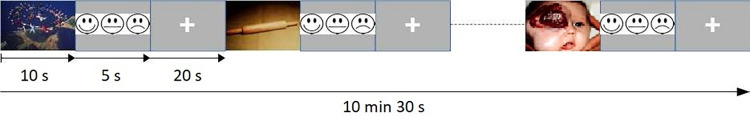
Emotional pictures viewing sequence scheme.

We selected eighteen pictures from the IAPS and the NAPS databases. Again, we excluded sexual-themed pictures because of frequent sexual trauma history in our study population. These pictures were divided into 3 groups according to their emotional valence and intensity: 6 images of positive valence and very high intensity (respectively rated > 7 and > 6.5); 6 images of negative valence and very high intensity (respectively rated < 3 and > 6.5); 6 images of neutral valence / low intensity (respectively rated between 4.5–5.5 and < 3.5) (Fig 3).

We will show these eighteen pictures one by one in a pseudo randomized order by ePrime software, in order to avoid two pictures of same valence to follow. Each picture will be shown for 10 seconds. After each picture, ePrime will show a smiling, a neutral and a sad face (respectively for positive, neutral and negative valences), at the same time and for 5 seconds; we will instruct each subject to indicate (with their right index finger) their own perception of the emotional valence of the picture. Again, this will be used to assess subjects’ focus on the task in the MRI. If the subject does not choose within time, the task will go on, but this will be taken into consideration for data analysis. Between each picture, a rest, a white cross on a grey screen for 20 seconds. Total sequence duration will be 10 minutes 30 seconds.

#### 3. Emotional Go / No go sequence

This task is to determine the importance of emotional distraction in an attention / inhibition task in the study population.

**Fig 4:**
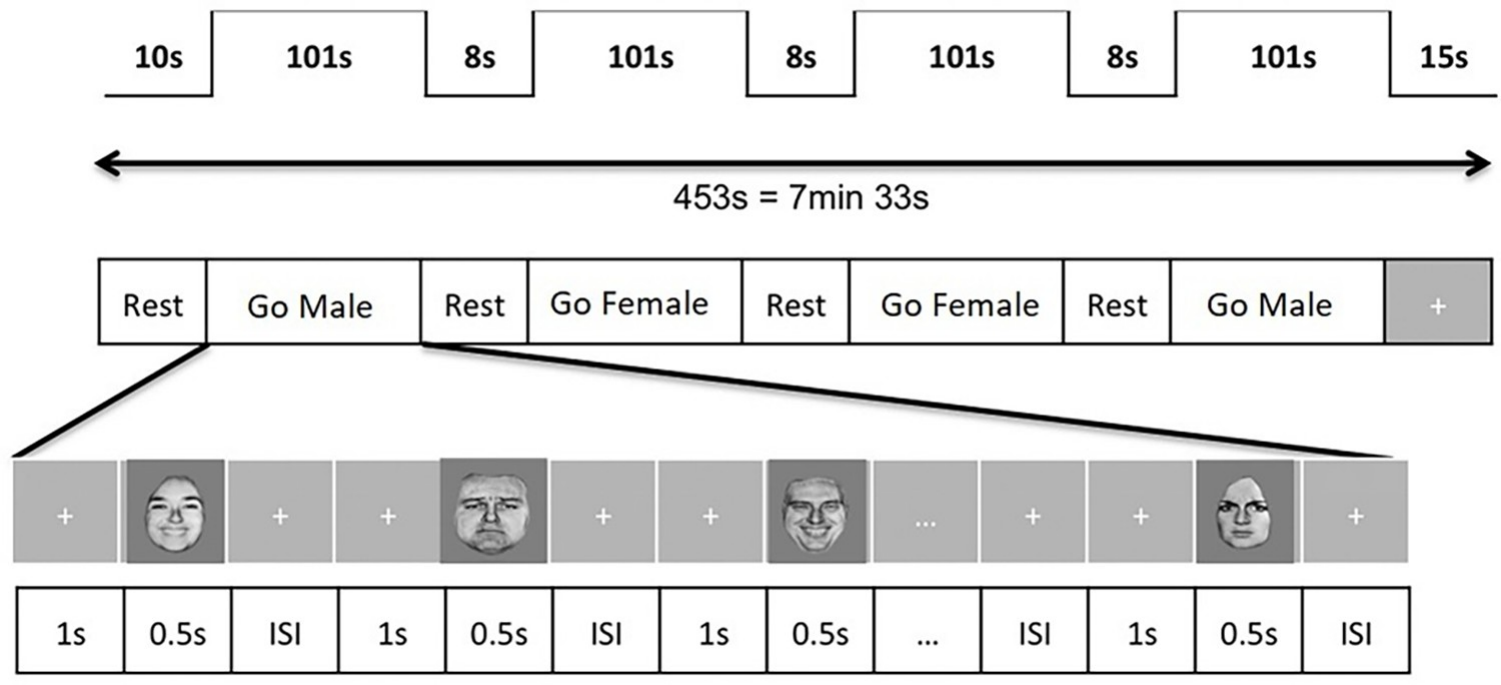
Emotional Go / No go sequence scheme. ISI: random time interval (2 to 6 seconds).

We will show faces of women and men, from the Ekman’s Pictures of Facial Affect collection [37], by turns of 500 milliseconds, each face displaying of different emotional valences (sadness, neutral, happiness). We will ask the participant to answer if she sees the face of a woman (during “Go Female” condition) or a man (during “Go Male” condition), and not to answer if she sees the face of a man (during “Go Female” condition) or a woman (during “Go Male” condition), regardless of the distractive emotion shown by the face. Each image will be separated by an interval of random time from 2 to 6 seconds. Each “Go Male” and “Go Female” condition series will last 101 seconds. Between each condition series of faces, a screen will display instructions for the next series for 8 seconds (e.g. “Click if the face is a man” for a “Go Male” condition sub-sequence). Each condition series will be processed twice, in alternation. The total duration of the sequence will be 7 minutes 33 seconds, including resting-states of 10 seconds at the beginning of the sequence (during which we will display the instructions of the first series) and 15 seconds at the end of the sequence (Fig 4).

#### 4. Resting state sequence

We will ask subjects to keep their eyes closed, without falling asleep, and to stay unmoving for 10 minutes.

#### 5. Positive emotional periodic oscillatory stimulation sequence

See subpart 1 above: Negative & positive emotional periodic oscillatory stimulation sequence, and Fig 2.

### Post-MRI examination

After giving the participant a 15-minute break, the investigating psychiatrist will interview her. She will be asked to rate subjectively the emotional valence and intensity of each one of the 18 pictures of the emotional pictures viewing sequence, using the SAM scale created by the IAPS designers. This rating is from 1 to 9 for valence and intensity and assesses the cognitive part of the emotional experience of the subject.

Finally, we will ask the subject for her general experience of the protocol and the psychiatrist will give her contact information in case she needs to talk again about it. We will not include these data in the analyses.

### MRI data analysis

Functional (expect to periodic oscillatory stimulations) and structural scans will be pre-processed and analysed using SPM software (Statistical Parametric Mapping, Version 12, Revision 7219, Wellcome Department of Cognitive Neurology, London, UK). Images will be automatically realigned to each other, and we will apply a correction for head-motion using a 6-parameters rigid body spatial transformation. Finally, we will normalize all images to the Montreal Neurological Institute (MNI) space, and smooth them using a Gaussian kernel ([6 6 6] FWHM) in order to improve signal to noise ratio. We will also use TAPAS PhysIO Toolbox [38] to denoise functional volumes. We will model the GE-EPI time-series using a first level general linear model [39] with regressors representing expected BOLD fMRI response to visual stimuli of task (colour arrangement or picture), separately for each session. Realignment parameters are due to be added in the regression models to remove specific activation due to head movement (translations “right”, “forward”, “up” and rotations “pitch”, “roll”, “yaw”) and we will use a 128 second high-pass filter to remove non-physiological slow signal shifts.

We will calculate contrasted parameter estimate images (con-images) for each subject. Such images describe brain activation for every visual stimulus (emotional and go / no go paradigms). We will use CONN toolbox (www.nitrc.org/projects/conn, RRID:SCR_009550) for resting state analysis [40]. This analysis is based on the identification of standard physiological functional neuronal networks (“default mode” network, motor network, sensory network, visual network, attention network). We will carry out this identification out without any preconception, by an analysis of independent components (MATLAB version 2017a and toolboxes). We will assess connectivity forces with a Pearson correlation coefficient between each region concerned by the identified and compared networks, using a graph of this connectivity.

The fMRI signal from periodic oscillatory stimulations will be compared using BrainVoyager QX (Version 2.8.0, Brain Innovation, Maastricht, Netherlands). Pre-processing will consist in a linear trend removal to exclude scanner-related signal, a temporal high-pass filtering applied to remove temporal frequencies lower than three cycles per run, and a correction for small interscan head movements by a rigid body algorithm rotating and translating each functional volume in 3D space. We will spatially align functional (unsmoothed) data with the high-resolution anatomical volume which will be previously aligned to the AC-PC plane (automatic co-registration in BrainVoyagerQX, adjusted manually). Subsequently, the pre-processed functional data will be exported and analysed using custom scripts written in MatLab. Following the same procedure as in [41], we will perform a Fast Fourier Transform (FFT) on the pre-processed BOLD time series to get the amplitude spectrum. Then, we will calculate the mean and standard deviations of the amplitude of the 40 frequency bins neighbouring the stimulation frequency (20 on each side, e.g. in [42, 43]). Lastly as in previous studies [44], we will calculate the signal-to-noise ratio (SNR) of the response at the stimulation frequency by converting its amplitude to a z-score using the computed amplitude mean and standard deviations from the neighbouring frequencies. This procedure will be applied to each voxel independently. With this procedure, we should obtain the magnitude of neural response at the stimulation frequency (0.111 Hz) in the negative and positive emotional periodic oscillatory stimulation sequences.

As in [41], we will define the activation and deactivation of the neural response using the phase of BOLD response at the stimulation frequency. In general, a positive phase value indicates increasing BOLD response amplitude after the onset of the target stimuli, whereas a negative phase value indicates decreasing BOLD response amplitude after the onset of the target stimuli. To account for individual time differences to reach maximum BOLD response amplitude, we will plot a histogram (20 bins) of phase values of all the voxels with a z-score above 3 and with only a positive phase value, for each individual. We will use the phase value of the histogram bin that has the largest count as the centre phase (φ) and define all the voxels with their phase values within φ ± π/2 as activations (+ sign) and voxels with their phase values outside of this window as deactivations (- sign). We will then apply the signs to the thresholded SNR (z-score) maps to obtain a final response map containing only voxels that have increased BOLD response (+ sign) to the presence of the target stimuli. We will use a conservative statistical threshold (uncorrected, p < 0.001) to define areas selective to positive/negative emotional images corresponding to z-values above *3*.*09*).

We will finally import the resulting functional activation maps back to BrainVoyagerQX for visualization.

### Statistical analysis

Statistical analysis will include a descriptive analysis of all participants and a dedicated treatment for fMRI images, as presented hereinabove.

We will describe quantitative variables by their means and standard deviations, quartiles as well as the maximum and minimum values and qualitative variables by the respective percentages of each element. The rating scales’ scores will be calculated according to their authors’ recommendations.

Functional MRI data will be initially processed at individual level, and then at group level. We will create a map of significant differences, with risk corrected at whole brain level, and with a p-value lower than 0.05. We will make separate individual statistical analysis, with a t-test comparing brain activity between emotional tasks and rest sequence, then a group analysis for each study group, and finally a statistical comparison between the three groups. Amongst other inter-group and potentially intra-group sub-comparisons, we will conduct a dedicated sub-analysis to investigate potential differences between PNES with and without PTSD.

### Patient and public involvement

This research is being conducted without patient involvement. Patients will not be invited to comment on the study design and will not be consulted to develop patient relevant outcomes or interpret the results. Patients will not be invited to contribute to the writing or editing of this document for readability or accuracy. Each participant is informed she can ask about study results (on a voluntary basis).

## Trial status & ethics

### Status of recruitment

To date, it is not complete for this study and inclusions are still going on.

### Version

This article describes EMOCRISES study protocol version 4, dated on January 21^st^, 2019. Important protocol modification will be communicated to the sponsor, CHRU of Nancy, or its Research and Innovation department direction.

### Data sharing statement

The database is being created. All data generated during this study will be made available via the CIC-IT, CHRU Nancy, Nancy–France, in accordance with the promoter protocol andEuropean General Data Protection Regulation. Data obtained from this study will be deposited at the CIC-IT of Nancy where they will be maintained for a minimum of 15 years.

## Ethics & dissemination

Ethics approval has been obtained on October 6th, 2016 from the institutional research ethics board (Comité de Protection des Personnes–CPP EST III) under the reference 16.10.01. EMOCRISES was accepted by the French National Medical Security Agency (ANSM) on November 2, 2016 under the reference 2016-A01295-46. The protocol and results will be published in peer-reviewed academic medical journals and disseminated to research teams, databases, specialised media and concerned patients’ organisations.

During the study, the investigator or any person designated in writing completes an electronic Case Report Form specific to the study for each participant subject, starting from source data. The identification of participant subjects is limited to a number assigned at inclusion. It will be a code composed as follows (first letter of the first name and first letter of the name), supplemented by a number attributed to the centre and to the inclusion. Only month and year of birth are collected. Images will be automatically transferred to a dedicated server in the Nancy CIC-IT using a secured/authenticated connection (HTTPS/FTPS). These data will be archived in the database ARCHIMED declared to the French authority (CNIL declaration number: 1410005). The investigator will be responsible for quality, accuracy and relevance of data collected in Case Report Forms. Data management for clinical and imaging data will be carried out by Nancy CIC-IT INSERM CIC 1433.

In accordance with the third paragraph of Article 56 of the Data Protection law, the presentation of the results of the data processing may under no circumstances allow the direct or indirect identification of the persons willing to be researched.

### Human faces shown during MRI sequences

As explained hereinabove, several sequences of the MRI acquisition involve showing series of human faces to the participants. Figs 3 and 4 show some of these faces and potential identifiable persons.

Concerning Fig 3, each image of the 18 we displayed during the visual emotional sequence, was selected either from the International Affective Picture System (IAPS) database or from the Nencki Affective Picture System (NAPS) database. As said in the IAPS designer’s message [45], this database “provides normative ratings of emotion (pleasure, arousal, dominance) for colour photographs that provide a set of normative emotional stimuli for experimental investigations of emotion and attention”. The baby face visible in the figure is one of these photographs. Most of the face pictures in these databases are of actors or models, some of them from other databases from different countries. Some other faces pictures are from media (mostly newspapers) but back in that time, they were selected with anonymity criterion. These databases were used in several studies around the world, including previous studies involving functional brain magnetic resonance imaging. They cannot lead to identify anypatient.

Concerning Fig 4, the images are from Dr Ekman’s Pictures of Facial Affect (POFA) collection. These images are all face pictures of volunteer or professional actors and models. Similarly to NAPS and IAPS, they were used in several studies around the world, including brain fMRI and they do not show patient’s faces.

## List of abbreviations

BOLD [contrast]: blood oxygen level dependent [functional MRI contrast]
CHRU: Centre Hospitalier Régional Universitaire (Regional University Hospital)
CIC-IT: Clinical Investigation Centre–Innovative Technology
CTQ: childhood trauma questionnaire
DES: dissociative experience scale
DSM-5: *Diagnostic and statistical manual of mental disorders*, 5^th^ edition
EEG: electroencephalography
GAPED: Geneva Affective Picture Database
HAMA: Hamilton anxiety rating scale
IAPS: international affective picture system
MADRS: Montgomery-Åsberg depression rating scale
MINI: mini international neuropsychiatric interview
MRI: magnetic resonance imaging
NAPS: Nencki affective picture system
PCL-5: PTSD check list, version 5 (i.e. according to DSM-5)
PNES: psychogenic non-epileptic seizures
PTSD: post-traumatic stress disorder
rTMS: repetitive transcranial magnetic stimulation
SAM: Manikin self-assessment [scale]
STAI: state-trait anxiety inventory
TAS20: Toronto alexithymia scale [in] 20 [items]
